# The enrichment ratio of atomic contacts in crystals, an indicator derived from the Hirshfeld surface analysis

**DOI:** 10.1107/S2052252514003327

**Published:** 2014-02-28

**Authors:** Christian Jelsch, Krzysztof Ejsmont, Loïc Huder

**Affiliations:** a CRM^2^, CNRS, Institut Jean Barriol, Université de Lorraine, BP 70239, 54506 Vandoeuvre les Nancy, France; b Faculty of Chemistry, Opole University, ul. Oleska 48, 45-052 Opole, Poland

**Keywords:** enrichment ratio, Hirshfeld surface analysis, crystal packing, fingerprint plots

## Abstract

The partitioning of space with Hirshfeld surfaces enables the analysis of fingerprint molecular interactions in crystalline environments. This study uses the decomposition of the crystal contact surface between pairs of interacting chemical species to derive an enrichment ratio. This quantity enables the analysis of the propensity of chemical species to form intermolecular interactions with themselves and other species. The enrichment ratio is obtained by comparing the actual contacts in the crystal with those computed as if all types of contacts had the same probability to form. The enrichments and contact tendencies were analyzed in several families of compounds, based on chemical composition and aromatic character. As expected, the polar contacts of the type H⋯N, H⋯O and H⋯S, which are generally hydrogen bonds, show enrichment values larger than unity. O⋯O and N⋯N contacts are impoverished while H⋯H interactions display enrichment ratios which are generally close to unity or slightly lower. In aromatic compounds, C⋯C contacts can display large enrichment ratios due to extensive π⋯π stacking in the crystal packings of heterocyclic compounds. C⋯C contacts are, however, less enriched in pure (C,H) hydrocarbons as π⋯π stacking is not so favourable from the electrostatic point of view compared with heterocycles. C⋯H contacts are favoured in (C,H) aromatics, but these interactions occur less in compounds containing O, N or S as some H atoms are then involved in hydrogen bonds. The study also highlights the fact that hydrogen is a prefered interaction partner for fluorine.

## Abstract 2

An enrichment ratio is derived from the decomposition of the crystal contact surface between pairs of interacting chemical species. The propensity of different contact types to form is investigated.

## 1. Introduction   

The involvement of functional groups in crystal packing contacts is widely investigated in the literature in order to better understand the principles of crystal formation. With the crystallographic analysis tools available using Hirshfeld surfaces, the crystal packing and contacts of chemical compounds can be studied extensively (Spackman *et al.*, 2002 ▶). Fingerprint plots can be made by evaluating the pairs of *d*_i_ and *d*_e_ distances from the surface to the nearest atom interior/exterior to the surface, respectively. *CrystalExplorer* software (McKinnon *et al.*, 2004 ▶; Wolff *et al.*, 2012 ▶) is now a tool widely used for visualizing and exploring intermolecular interactions in crystals. The program notably enables fingerprinting of intermolecular interactions by pairs of chemical species. In this manner different interaction types such as hydrogen bonding, van der Waals contacts, C—H⋯π and π⋯π stacking can be identified in the fingerprint plots. The *d*_norm_ quantity, which was introduced by McKinnon *et al.* (2007 ▶), is negative where contacts are shorter than the van der Waals separation, and positive for longer contacts. This quantity plotted on a Hirshfeld surface provides a very informative tool to analyze crystal packings.

The *CrystalExplorer* software is also a powerful tool for the visualization and characterization of voids in crystalline materials (Turner *et al.*, 2011 ▶). When electrostatic potentials of molecules are mapped onto their Hirshfeld surfaces and displayed within a crystal packing diagram, the molecular contacts can be analyzed in terms of the electrostatic complementarity of touching surface patches (Spackman *et al.*, 2008 ▶).

In the current paper a new descriptor is proposed to analyze contacts in molecular crystals. The surface contact data derived from the Hirshfeld surface analysis are used to derive enrichment ratios which relate to the propensity of pairs of chemical species to form crystal-packing interactions.

Several series of compounds composed of the same chemical species were analyzed to identify trends in crystal-packing contacts. A selection of CIF files containing the structural coordinates of the molecules were retrieved from the Cambridge Structural Database (CSD; Allen, 2002 ▶). For the crystal structures collected, the contacts are analyzed in terms of chemical types involved at the Hirshfeld surface. The propensity of two chemical species to be in contact are analyzed and discussed for several sets of crystal structures containing a limited number of elements.

## 2. Materials and methods   

### 2.1. Hirshfeld surface and fingerprint plots   

Hirshfeld surfaces were introduced by Spackman & Byrom (1997 ▶) to partition the space in molecular crystals for electron-density integration purposes. The Hirshfeld surface is a region of crystal space around the molecule that is defined by *W*(**r**) = 0.5, where *W* is a weight function derived from the atomic electron densities ρ_i_(**r**)
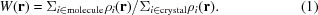
Thus, the Hirshfeld surface can be considered as the frontier between regions where the electron distribution is dominated by the contribution of the reference molecule (interior) and of the neighbouring molecules in the crystal (exterior).

The program *CrystalExplorer* (Wolff *et al.*, 2012 ▶) enables the analysis and visualization of crystal contacts through the Hirshfeld surface. Using the CIF files *CrystalExplorer* can compute the Hirshfeld surface and then the fingerprint plots. The contact surface decomposition in these plots by specific pairs of chemical elements is the interesting resulting information. This allows the calculation of derived properties such as the percentage of surface that involves a particular element.

### 2.2. Definition of the enrichment ratio   

The percentage of contacts between one (*X*⋯*X*) or two (*X*⋯*Y*) chemical elements in a crystal packing is information given by *CrystalExplorer* which can be used to indirectly calculate the enrichment ratios.

The proportion of Hirshfeld surface contacts involving the (*X*,*Y*) pair of elements is referred to as *C*_*XY*_. The proportion *S*_*X*_ of chemical type *X* on the molecular surface is obtained by the summation
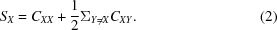
The value *C*_*XY*_ includes both *X*⋯*Y* and *Y*⋯*X* contacts in the *S_X_* sum, where the first and second atoms are interior and exterior to the Hirshfeld surface. The factor ½ relates to the fact that *C*_*XY*_ contributes to both *S*_*X*_ and *S*_*Y*_ summations. The summation of all the surface proportions is equal to unity
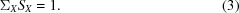



The ratio of random contacts *R_XY_* between the two chemical elements *X* and *Y* is then introduced. The *R*_*XY*_ values are defined as if all contact types *X*⋯*Y* in the crystal packing were equi-distributed between all chemical types and are obtained by probability products
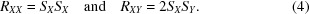
The *R*_*XY*_ proportion again includes both *X*⋯*Y* and *Y*⋯*X* contacts, which explains the presence of factor 2 in this definition.

The sum of all random contact proportions also yields unity, by definition
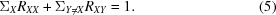
The enrichment ratio *E*_*XY*_ of a pair of elements (*X*,*Y*) is then defined as the ratio between the proportion of actual contacts in the crystal and the theoretical proportion of random contacts
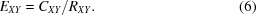
The enrichment ratio is expected to be generally larger than unity for pairs of elements which have a high propensity to form contacts in crystals, while pairs which tend to avoid contacts with each other should yield an *E* value lower than unity.

### 2.3. Selection of molecules   

The enrichment ratios *E*_*XY*_ were computed for sets of molecules belonging to several classes of molecules and were analyzed as a function of *S*_*X*_ values. In order to limit the number of *E*_*XY*_ values and *S*_*X*_ variables present in the contacts data, compounds containing only two or three different chemical elements were selected and grouped in their respective set: CH, CHN, CHS, CHO, CHF.

Aliphatic molecules are defined here as being without aromatic rings and are generally devoid of double or triple bonds, except carbonyl and carboxylate groups which were accepted. These compounds generally have a large number of H atoms on their surface (*S*_H_ large), while C atoms are rarely present at the molecular surface (*S*_C_ ≃ 0), as they generally form four bonds with other atoms.

On the other hand, ‘aromatic sets’ of molecules were selected for having mainly planar rings and double bonds. In these molecules, carbon is significantly present on the molecular surface (*S*_C_ > 0). Due to this major difference in the molecular surface composition, aliphatic and aromatic molecules were separated into different groups. The molecules were selected in the CSD in order to avoid ambiguities and were either purely (or mostly) aromatic or largely aliphatic.

## 3. Results and discussion   

### 3.1. Example of enrichment ratio calculation   

Privileged and disfavoured contacts from a chemical elements point of view can be highlighted in a crystal structure *via* the enrichment ratios. An example of a calculation for the coumarin 102 molecule (C_16_H_17_NO_2_) is given in Table 1 ▶. This molecule is composed of two aromatic cycles (one of which is heterocyclic), two aliphatic cycles and a methyl group. More than three quarters of the molecular surface is generated by H atoms, while the two other significantly contributing elements are carbon and oxygen, slightly above 10%. C atoms are exposed to the molecular surface only in molecules where they form fewer than four covalent bonds, which is the case in aromatic rings and more generally for C*sp* ^2^ atoms. Among the actual contacts, H⋯H accounts for more than half of the interaction surface, while C⋯H and O⋯H contacts are the only other significant contributions (> 15%). C⋯H contacts represent C—H⋯π interactions on the two sides of the aromatic cycles and O⋯H—C contacts are weak hydrogen bonds, in the case of coumarin 102.

The list of enrichment ratios highlights the O⋯H contacts (*E*_OH_ = 1.24, Table 1 ▶) which turn out to be favoured in the crystal packing, notably the O=C oxygen atoms form three weak hydrogen bonds of the type O⋯H—C. The C⋯H and H⋯H contacts are, respectively, slightly enriched and disfavoured (*E*_CH_ = 1.04 and *E*_HH_ = 0.96). The enrichment ratios of other types of contacts shall be analyzed cautiously as resulting from quotients of two small numbers. Their contributions are very small, below 2.6% if equiprobable contacts *R*_*XY*_ are considered. It can still be observed that C⋯C contacts are favoured (*E*_CC_ = 1.5) and that O⋯O contacts are completely avoided in coumarin 102 with *E*_OO_ = 0.0.

### 3.2. CH aromatic compounds   

**Figure 1:**
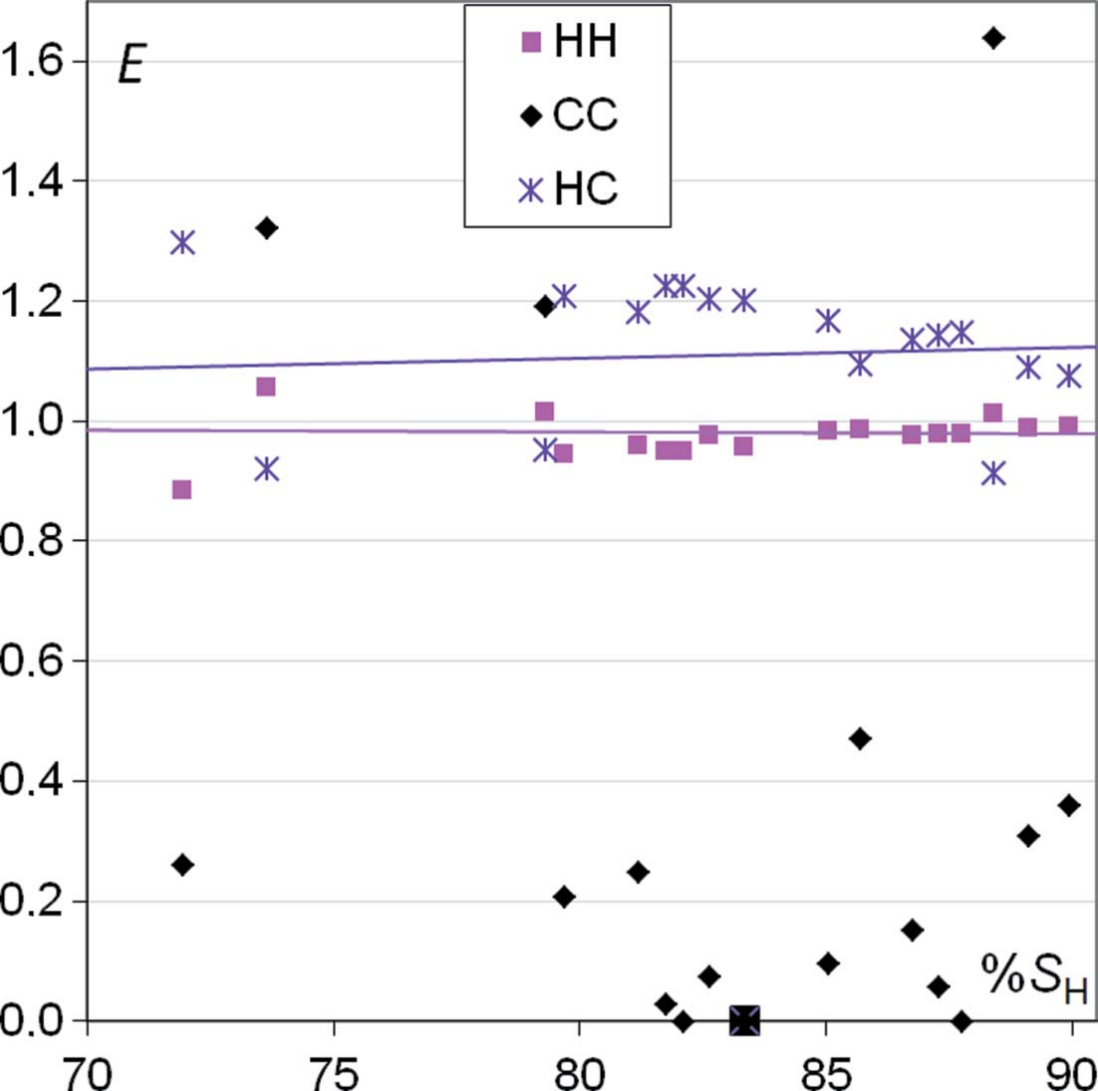
Contact enrichment ratios in crystals of aromatic CH compounds as a function of hydrogen proportion on the Hirshfeld surface. The case of benzene is highlighted.

The crystal contacts of a series of aromatic compounds containing only H and C atoms are analyzed in Fig. 1 ▶. The crystal packings of polynuclear aromatic hydrocarbons have been classified as stack, layer, glide or herringbone and their occurrences have been rationalized on the relative importance of C⋯C and C⋯H interactions, which depend on the H/C composition and positioning in the molecule (Desiraju & Gavezzotti, 1989 ▶).

The H⋯H contacts appear with enrichments ratios close to unity and necessarily constitute most of the interaction surface as *S*_H_ is larger than 65% in this set of molecules. Matta *et al.* (2003 ▶) analyzed ubiquitous hydrogen⋯hydrogen bonding by the quantum theory of atoms in molecules and found that they are stabilizing interactions in crystal packings. Interestingly, H⋯H contacts were found to become extremely prominent at high pressures in surface fingerprint plots (of compounds containing C, H, N and O atoms) and did not appear to compress below a H⋯H distance of 1.7 Å in a recent study (Wood *et al.*, 2008 ▶).

On the other hand, H⋯C contacts are generally slightly favoured in a sample of CH aromatic molecules with an average *E*_HC_ value of 1.1. The C⋯C contacts are in most cases very disfavoured with *E*_CC_ < 0.5. The case of the benzene crystal with extensive C—H⋯π stacking is characteristic of the CH aromatic class of compounds, as it displays a *E*_CC_ value of zero (Fig. 1 ▶), *E*_CH_ = 1.21 and *E*_HH_ = 0.96.

However, three compounds relatively rich in carbon on the Hirshfeld surface (*S*_c_ > 0.24) show slight *E*_CC_ enrichments around 1.2. These molecules have large aromatics parts composed of three or four adjacent rings and are involved in π⋯π stacking.

The geometry of aromatic interactions has been investigated notably by Hunter (1994 ▶; Hunter *et al.*, 2001 ▶), who explained that π⋯π stacking can be associated with repulsive electrostatic interactions and is therefore less favourable than could be expected on the basis of the van der Waals interactions. Due to π-bonding in aromatic rings, the deformation electron-density peaks on the C—C bonds are elongated in the direction perpendicular to the aromatic ring and a global quadrupole is generated in that direction. On the other hand, π-facial hydrogen bonds (C⋯H interactions) are favoured by the charge distribution.

### 3.3. CHN aromatic compounds   

**Figure 2:**
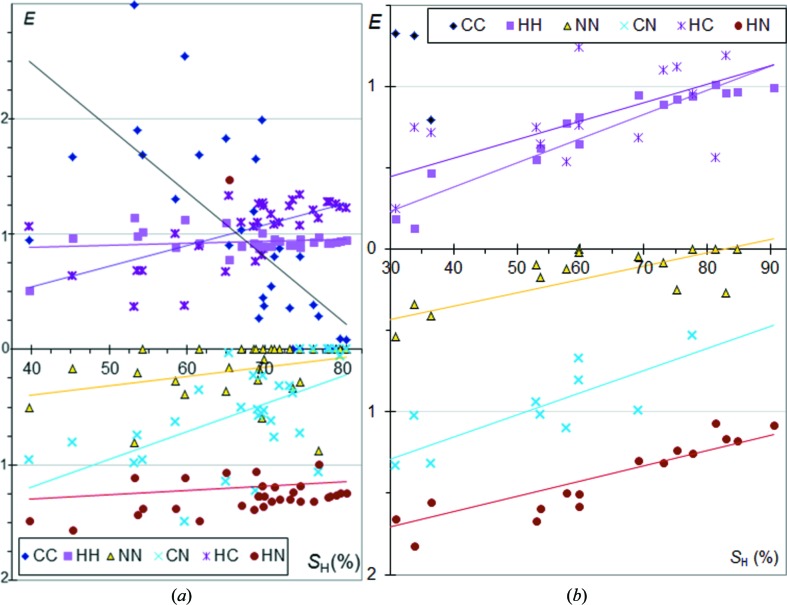
Contact enrichment ratios in crystals of CHN compounds as a function of hydrogen proportion on the Hirshfeld surface. (*a*) Aromatics; (*b*) CH aliphatics containing nitrile groups.

Enrichment ratios of the different types of contacts are shown for a sample of aromatic CHN molecules in Fig. 2 ▶. The set of CHN aromatic molecules is composed of molecules with (C,N) heterocycles, but also contains nitrile and planar N atoms (—CN, —NN, —NH_2_). Fig. 2 ▶ and several subsequent figures display the different enrichment ratios as a function of the molecular surface percentage due to H atoms. This element was chosen instead of others because it shows a large range of surface proportion values, making the graphs more straightforward and easier to read. Moreover, hydrogen is the most frequent chemical type occurring at the surface of organic compounds, therefore the proportion of H surface is an important indicator. Other representations of the data, with a different quantity chosen for the *X* axis (for example *S*_N_), are shown in the supporting information and yield another perspective and additional information on the crystal contacts.

In Fig. 2 ▶ the enrichments of the H⋯H contacts are stable around unity, generally slightly below. The H⋯N contacts, which can be N—H⋯N or C—H⋯N hydrogen bonds or H⋯π contacts, are favoured as they consistently show enrichments between 1.0 and 1.6, but they are slightly more enriched for molecules with the lowest content of hydrogen. The N⋯N contacts generally show the lowest enrichment with all values below 0.9 and half of the compounds actually displaying no N⋯N contact.

On the other hand, C⋯C contacts show a wide range of enrichments from 0 to 3. The general tendency for *E*_CC_ is to be high for molecules with low hydrogen content, but small, even reaching zero values, for compounds rich in hydrogen. On the contrary, H⋯C interactions are more likely to occur when *S*_H_ is high. As a consequence, C—H⋯π and N—H⋯π interactions are found to be favoured when H atoms are highly available, but replaced by some C⋯C contacts, which are π stackings, when H atoms are less abundant.

The graph of *E* ratios as a function of *S*_N_ values (Fig. S1) shows that, in CHN aromatic molecules, the propensity for H⋯H and H⋯N interactions remains stable. On the other hand, H⋯C contacts are more enriched for compounds with poor nitrogen content and the *E*_HC_ coefficient is then similar to that found for CH aromatic compounds (Fig. 1 ▶). Compounds rich in nitrogen display generally lower *E*_HC_ values as more H atoms are involved in H⋯N contacts which can be hydrogen bonds. N⋯N contacts are disfavoured compared with C⋯N interactions which have themselves *E*_CN_ values generally lower than unity. Both contact types show increasing enrichments as nitrogen becomes more abundant on the molecular surface. This is to be related to the higher propensity of heterocycles to form π stacking as δ^+^ and δ^−^ polarized atoms can be interacting partners (Salonen *et al.*, 2011 ▶).

### 3.4. CHN aliphatic nitrile compounds   

A series of CH aliphatic molecules containing additional nitrile groups was also analyzed. The proportion of carbon on the Hirshfeld surface is generally small in these compounds and originates from the nitrile groups. The trends are similar to CHN aromatic molecules for H⋯C, C⋯N and N⋯N contacts (Fig. 2 ▶ *b*). *E*_HN_ enrichments are found to vary more with the *S*_H_ proportion.

The behaviour of H⋯H contacts is different as *E*_HH_ is found to decrease significantly below unity for molecules with poor hydrogen content. There is a very clear depletion of H⋯H contacts presumably due to the involvement of H atoms in C—H⋯N hydrogen bonds. For compounds with a very high hydrogen content on the surface, the enrichment *E*_HH_ tends, by definition, towards a value close to 1.

The C⋯N contacts tend to be enriched for molecules with a low content in hydrogen but are very impoverished when *S*_H_ is large, presumably as the N atoms are then involved in C—H⋯N attractive hydrogen bonds.

The same data viewed as a function of *S*_N_ show systematically significant slopes for the fitted curves of the enrichment ratios (Fig. S1). A global increase of H⋯N and C⋯N contact enrichments is observed as nitrogen becomes more abundant. The N⋯N contacts also show an increase of enrichments with *S*_N_, but the values always stay well below unity. A major difference with CHN aromatic compounds is that *E*_HN_ and *E*_HH_ values are not stable but increase/decrease significantly, respectively, with *S*_N_.

**Figure 3:**
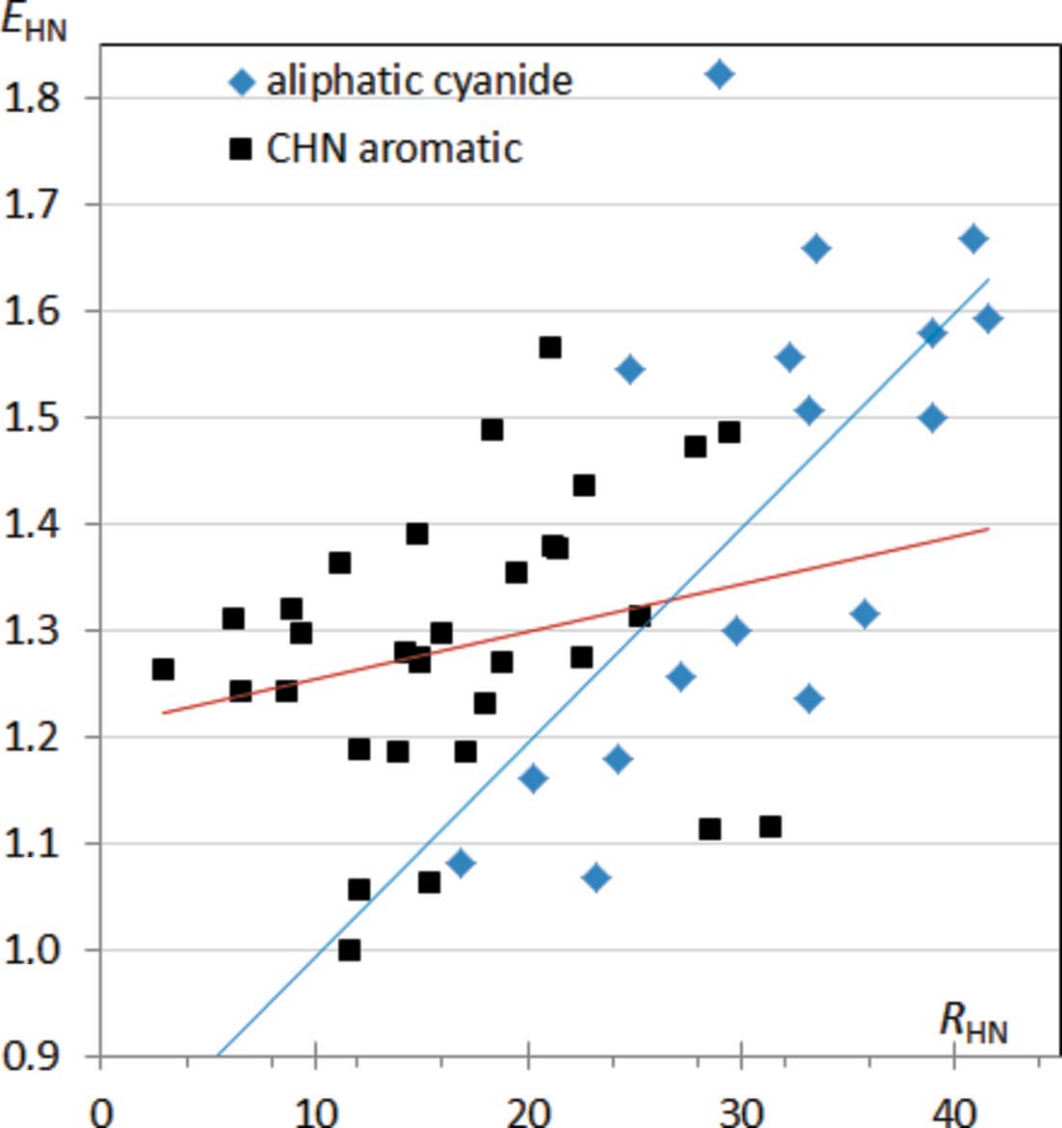
Enrichment of H⋯N contacts in CHN compounds as a function of the proportion *R*_HN_ (%) in CHN-type molecules.

Enrichment ratios can also be analyzed as a function of other variables, such as the proportion of random (or effective) contact surface in the crystal packing. Fig. 3 ▶ shows the enrichment ratio of H⋯N contacts as a function of *R*_HN_ random contact proportion, which depends on the content of C and N atoms on the Hirshfeld surface. The *E*_HN_ enrichments generally increase with *R*_HN_ for both types of CHN molecule. However, aliphatic nitrile compounds show a greater slope of *E*_CN_ *versus R*_CN_ compared with aromatic molecules.

### 3.5. CHO compounds   

**Figure 4:**
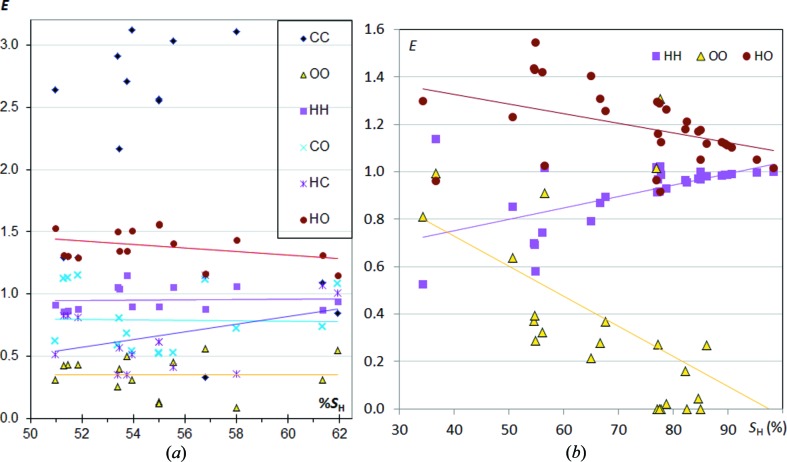
Contact enrichment ratios in crystals of CHO compounds as a function of hydrogen on the Hirshfeld surface: (*a*) aromatics; (*b*) aliphatics.

In CHO aromatic compounds (Fig. 4 ▶ *a*) the O⋯O contacts are quite impoverished, generally with *E*_OO_ below 0.5. On the other hand, H⋯O contacts with *E*_HO_ in the [1.2, 1.6] range are, as expected, quite enriched due to the formation of energetically favourable hydrogen bonds. The enrichment of H⋯H contacts shown as a function of *S*_H_ remains stable around unity, but decreases slightly when *S*_O_ augments (Fig. S2), due to competition with H⋯O hydrogen bonds.

**Figure 5:**
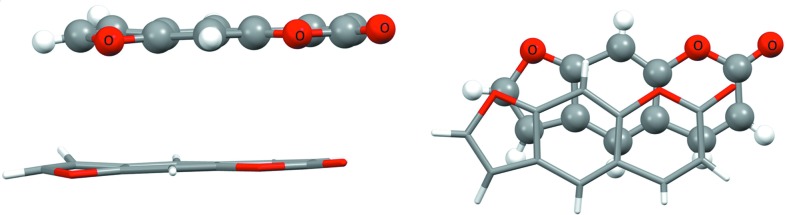
Example of furo[3,2-*g*]coumarin, a CHO aromatic molecule with heterocycles displaying π⋯π stacking. The two figures showing a dimer are rotated by 90° around the horizontal direction. The two interacting molecules are related by translation along the *c* axis in the triclinic *P*1 space group.

The proportion of carbon on the Hirshfeld surface for this set of molecules is between 16 and 24%. C⋯O and H⋯C contacts are generally impoverished with average values of 〈*E*_CO_〉 = 0.8 and 〈*E*_HC_〉 = 0.7, which are below unity. Despite a few exceptions, the C⋯C contacts are the most enriched ones, with many molecules overcoming the 2.5 mark, especially the compounds low in hydrogen at the surface. The *E*_CC_ enrichment is generally higher in CHO (and CHN) compounds compared with pure aromatic CH hydrocarbons. This is in accordance with reports that π-stacking between aromatic heterocycles is favourable in face-to-face geometry where there is a good superimposition of the positively and negatively charged atoms (Salonen *et al.*, 2011 ▶; Martinez & Iverson, 2012 ▶). An example of CHO aromatic compounds displaying extensive π⋯π stacking is shown in Fig. 5 ▶ for furo[3,2-*g*]coumarin (Bideau *et al.*, 1979 ▶), a molecule which is also present in Fig. 4 ▶(*a*) with *S*_H_ = 53% and *E*_CC_ = 2.91. In π⋯π stacking the O atoms interact with positively charged C atoms which are themselves covalently bonded to O atoms. In the other directions, the interactions are of the type H⋯H and H⋯O, with one strong C—H⋯O=C hydrogen bond.

Globally the scatterplots for CHO and CHN molecules (Figs. 2 ▶ and 4 ▶) show many similarities if nitrogen and oxygen are considered as equivalent, both chemical types being electronegative and prone to make polar contacts such as hydrogen bonds. When the contacts in CHO aromatics are analyzed as a function of *S*_O_ (Fig. 2 ▶), the proportion of oxygen on the molecular surface varies over a narrow range (21–32%). The different enrichments show generally stable values, with no clear increasing or declining trends, except for H⋯H interactions which decrease with *S*_O_. The equivalent graph (*S*_O_, *E*) for CHO aliphatics is redundant: as C atoms are scarcely present at the molecular surface, the proportions *S*_H_ and *S*_O_ are nearly complementary (*S*_H_ + *S*_O_ ≃ 1).

In CHO aliphatics (Fig. 4 ▶ *b*), some molecules have a high proportion of H atoms on their surface. The most striking trend appearing with the *S*_H_ increase is the convergence of both H⋯O and H⋯H contacts to enrichments close to 100%, the first one by decreasing and the second one by increasing. The main difference with CHO aromatics is that *E*_OO_ strongly decreases when the proportion of hydrogen on the molecular surface decreases. When H atoms are abundant, O⋯O contacts are not favoured as O atoms tend to be involved in O⋯H hydrogen bonding.

**Figure 6:**
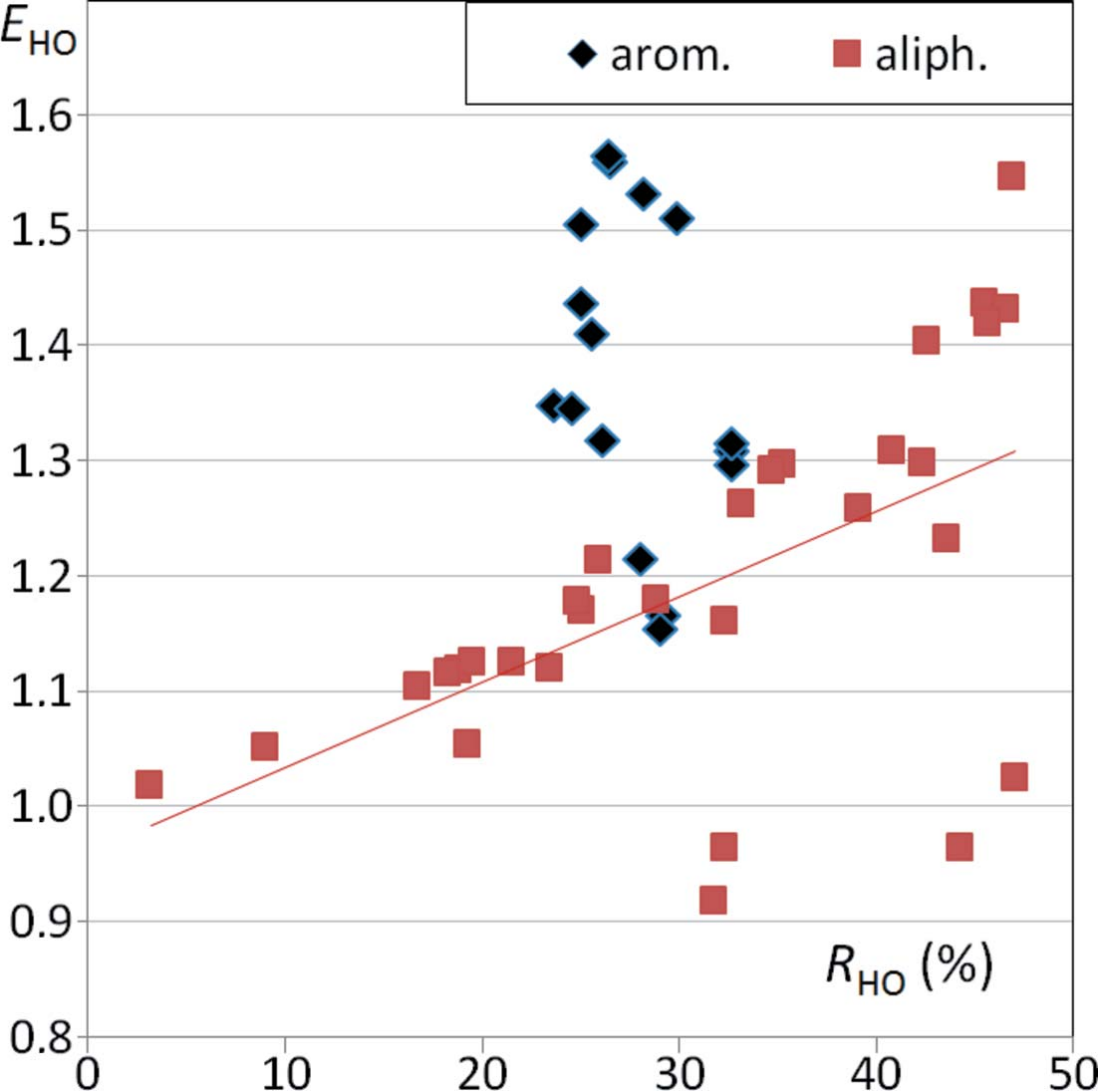
Contact enrichment ratios *E*_HO_ in crystals of CHO compounds as a function of *R*_HO_ random contact surface.

The enrichment ratios of H⋯O contacts are displayed as a function of *R*_HO_ in Fig. 6 ▶. These contacts are generally enriched, but there are a few exceptions with *E*_HO_ slightly smaller than 1. The graph highlights the fact that for CHO aliphatics, the H⋯O contacts have an increased likelihood to form when the molecules are rich in both oxygen and hydrogen (large *R*_HO_ values). The *R*_HO_ values are restricted to a small range of values in the sample of studied CHO aromatic molecules, but the *E*_HO_ enrichments are comparatively higher than for aliphatic molecules.

In both subsets of CHO compounds, the O⋯O contacts are globally impoverished, but their likelihood to occur increases strongly as oxygen becomes more abundant at the molecular surface (Fig. S3 shows *E*_OO_ as a function of *S*_O_). The *E*_OO_ ratios are very small, close or equal to zero, in CHO aliphatic molecules when the surface contact in poor in oxygen (*S*_O_ < 20%). On the other hand, O⋯O contacts can have *E*_OO_ ratios close or even larger than one for some molecules richer in oxygen. Aromatic molecules show a narrow range of *S*_O_ values, but the corresponding *E*_OO_ ratios are comparable to those of aliphatic molecules with a similar oxygen content on the surface. Oxygen–oxygen contacts in crystals have been discussed from an energetic point of view (Gavezzotti, 2010 ▶); they generally do not occur in a standalone way but are secondary to stronger interactions.

### 3.6. CHS compounds   

**Figure 7:**
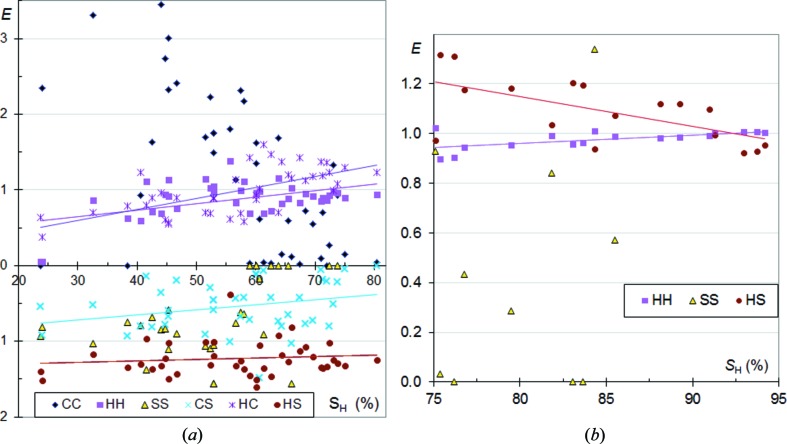
Contact enrichment ratios in crystals of CHS compounds as a function of % of hydrogen on the Hirshfeld surface: (*a*) aromatics; (*b*) aliphatics.

The set of CHS aromatic compounds selected contain 10–32% carbon and 3–66% sulfur on the molecular surface. The H⋯S contacts have generally stable *E*_*S*_ values in the [1.0, 1.5] interval, generally above unity as sulfur is a weak hydrogen-bond acceptor (Fig. 7 ▶ *a*). The S⋯S contacts are actually totally absent for several compounds rich in hydrogen or low in sulfur (Fig. S4). For CHS compounds with a lower content of hydrogen on the surface, the S⋯S contacts can be moderately enriched, as *E*_*S*_ values are between 0.6 and 1.6. On the other hand, C⋯S contacts are generally under-represented with *E*_CS_ ratios taking, in general, values between 0 and 1.

On the contrary, both H⋯C and H⋯H contacts tend to increase with *S*_H_; these contacts have enrichment ratios which are generally between 0.5 and 1.4. C⋯C contacts often show high enrichments between 1.5 and 3.5 for molecules with low hydrogen content, while molecules with a high *S*_H_ proportion have a much lower propensity to form C⋯C interactions, with *E*_CC_ close to zero for several crystal packings. This is to be related to the higher propensity of C atoms in contact with hydrogen when *S*_H_ is high. *E*_CC_ ratios larger than 2.5 are related to molecules with carbon content *S*_H_ < 17% (or *R*_HH_ < 2.8%) on their surface and their significance is limited as issued from ratios with small divisors.

Similarly, H⋯H interactions tend to be impoverished for compounds poor in hydrogen, or rich in sulfur (Fig. S4). H⋯S interactions show *E*_HS_ values around 1.2 for compounds with low sulfur content, *i.e.* which contain mostly H and C; this was also observed for CHN and CH compounds. The graph of contact enrichment as a function of *S*_S_ (Fig. S5) shows a strong increase of *E*_CC_ and *E*_*S*_ with the proportion of sulfur at the molecular surface, while *E*_HH_ and *E*_HC_ tend to decrease.

In CHS aliphatic molecules, the proportion of Hirshfeld surface related to C atoms is generally zero or very small, therefore contacts involving C atoms are not shown in Fig. 7 ▶(*b*). For molecules rich in hydrogen on the surface (*S*_H_ > 88% implying *S*_S_ < 12%), *R*_*S*_ values are consequently very small and the *E*_*S*_ data are discarded in the graph.

H⋯H and H⋯S contacts show the same behaviour as for aromatic CHS compounds and their enrichment ratios tend to unity when the proportion of H atoms on the Hirshfeld surface becomes large. The S⋯S contacts show a large range of *E*_*S*_ enrichments from 0.0 to 1.3.

### 3.7. CHF compounds   

Very few purely aliphatic CHF-type molecules were found, therefore, the analysis was made on a sample containing molecules with aromatic rings which were more abundant in the CSD. In addition, a few relatively small molecules (up to four C atoms) containing C=C double bonds were also considered, which similarly display carbon on their surface.

**Figure 8:**
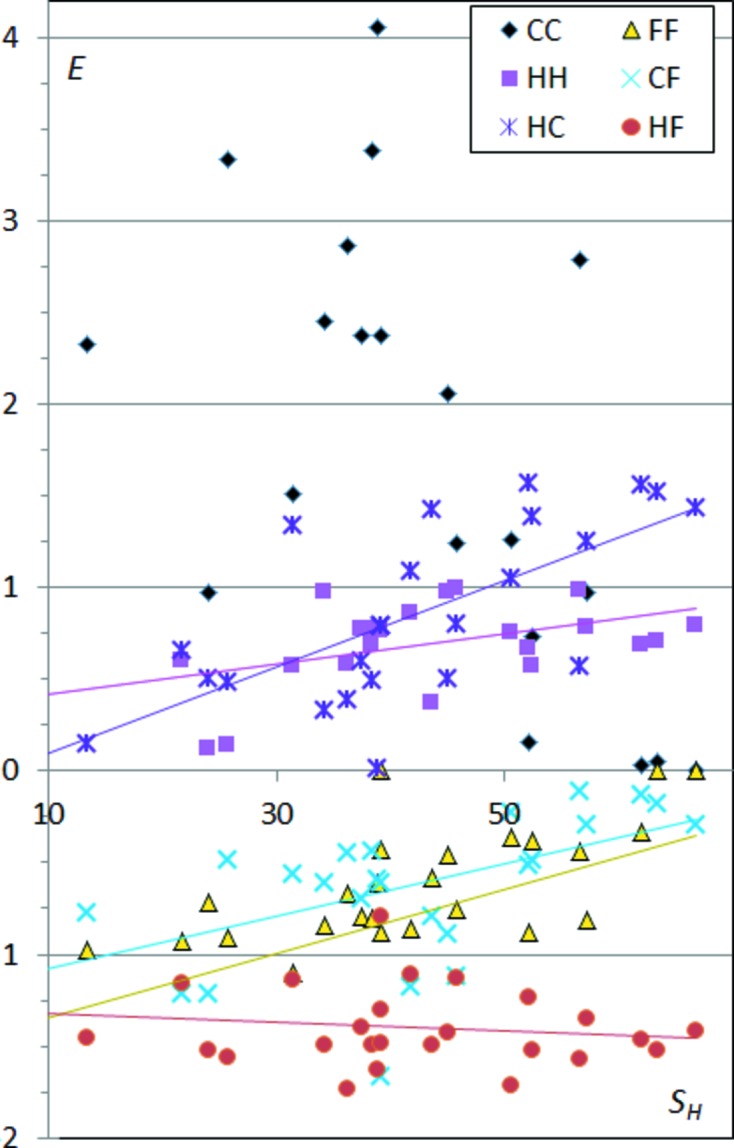
Contact enrichment ratios in crystals of CHF compounds as a function of % of hydrogen on the Hirshfeld surface.

Interactions involving organic (C—F) fluorine are generally considered as weak, although fluorine is electronegative and displays an electron accumulation of torus shape around the C—F axis (Bach *et al.*, 2001 ▶). The enrichment of the F⋯F contacts is generally lower than unity (Fig. 8 ▶) and is globally decreasing with *S*_H_ from unity to values as low as zero. The C⋯F contacts show a similar trend; the *E*_CF_ ratios are generally lower than one and are steadily decreasing as the content of hydrogen becomes high. This is to be related to the fact that C—F⋯π interactions occur mostly in perfluorinated aromatic rings which are electron depleted (Reichenbächer *et al.*, 2005 ▶; Berger *et al.*, 2011 ▶). This last statement is clearly confirmed as *E*_FC_ ratios increase as compounds become rich in fluorine (Fig. S5).

The H⋯F contacts appear with high enrichment values stable around 1.5 ± 0.4, indicating that hydrogen is a preferred partner in contacts made by F atoms, as the two atom types are attractive from an electrostatic point of view. The role of organic fluorine in crystal engineering and notably the occurrence of H⋯F and F⋯F interactions in crystals was recently reviewed. Choprah & Row (2011 ▶) and Thalladi *et al.* (1998 ▶) studied C—H⋯F interactions in the crystal structures of several fluorobenzenes. Comparing the crystal packings of similar compounds they found that C—H⋯F interactions resemble C—H⋯N and C—H⋯O interactions and can be considered as weak hydrogen bonds. In molecules with a high fluorine content, C—H groups have increased acidity and the C—H⋯F interactions become stronger. They also showed that the C—F group prefers to form C—H⋯F interactions rather than F⋯F contacts, which is in accordance with the related enrichment ratios found in this study.

The H⋯H contact enrichment ratios globally increase in CHF compounds with the percentage of H at the molecular surface, but remain generally lower than unity (Fig. 8 ▶). *E*_HH_ can be quite low for CHF compounds low in hydrogen due to competition with H⋯F contacts.

The C⋯C contacts are, in many cases, very enriched. A few compounds with high *S*_H_ display, on the contrary, an absence or strong under-representation of these contacts and correlatively H⋯F are over-represented.

The same data displayed as a function of *S*_F_ show that the F⋯F contacts are generally impoverished, but increase and tend to a unitary enrichment for molecules with a large fluorine content. The C⋯F contacts show a similar increasing trend with *S*_F_. H⋯F contacts are again stable around *E*_HF_ = 1.5, while H⋯H contacts have a decreasing likelihood of occuring for fluorine-rich molecules as H atoms are attracted by F atoms.

### 3.8. C⋯C contacts   

**Figure 9:**
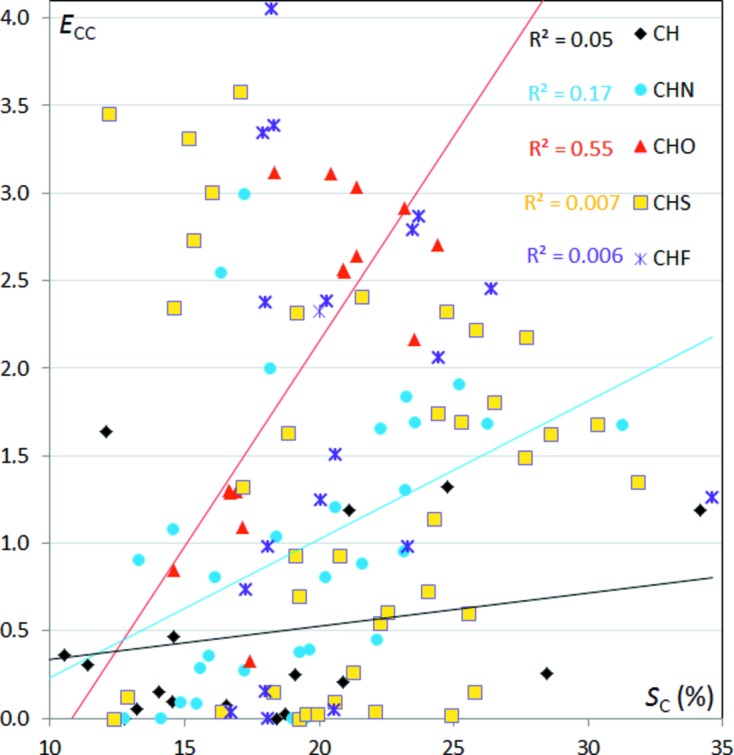
Contact enrichment ratios *E*_CC_ *versus* proportion of carbon *S*_C_ in the different sets of aromatic compounds.

The C⋯C interactions were further investigated as they show the highest enrichment values. The *E*_CC_ ratios are displayed as a function of *S*_C_ in the different sets of aromatic compounds in Fig. 9 ▶; they show a wide range of values from 0 to 4. The *E*_CC_ values increase globally with *S*_C_ for CHO compounds and the two variables are 55% correlated. The same trends hold for CHN compounds, but the slope of the linear fit and the correlation coefficient are reduced. For CH, CHS and CHF compounds, the *E*_CC_ and *S*_C_ variables show no correlation. The *E*_CC_ ratios larger than 3 originate from molecules with less than 21% carbon on the surface, *i.e.* the divisor *R*_CC_ < 4.4% is small in the enrichment quotient.

**Figure 10:**
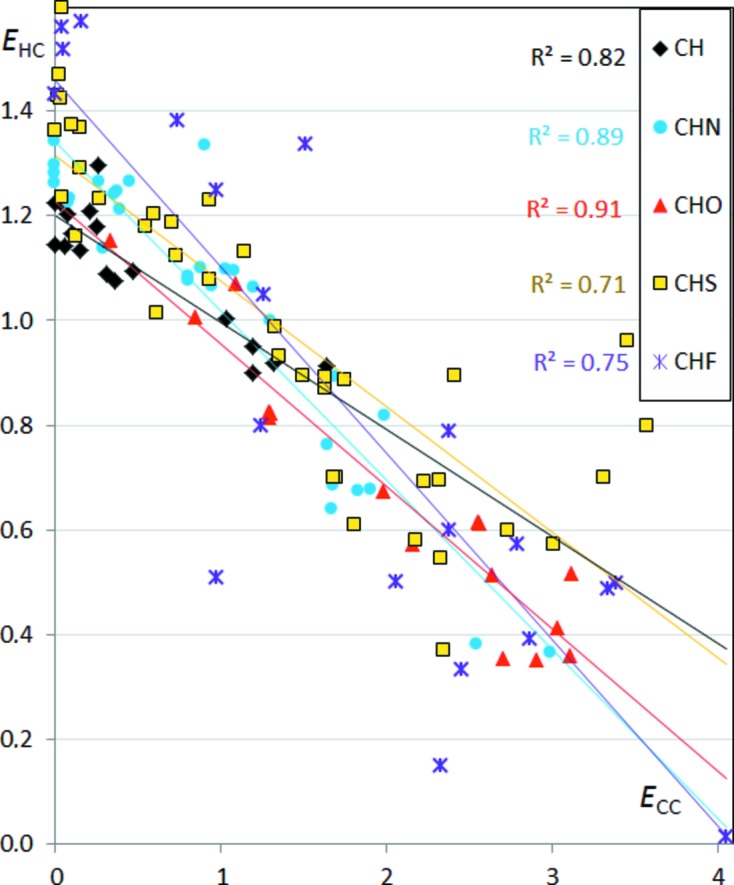
Contact enrichment ratios *E*_HC_ *versus E*_CC_ in the different sets of aromatic compounds.

Enrichment values related to different interactions can also be analyzed together. The graph of the enrichment ratios *E*_CC_ *versus E*_HC_ (Fig. 10 ▶) shows that the two variables are in all cases highly anti-correlated (|ρ| > 71%). This suggests that C⋯C and H⋯C interactions are strongly in competition with each other in the crystal contacts. The highest anti-correlations are found for the CHO and CHN compounds, *i.e.* which contain the polar atoms O or N.

## 4. Conclusions   

The contacts between chemical species were analyzed in several sets of crystal structures containing at most three different chemical types. The percentage of the surface of different elements which compose each molecule was derived from the analysis with *CrystalExplorer*. Subsequently, contact enrichment ratios were computed for all pairs of chemical elements. The fraction of enrichment is obtained by dividing the effective surface contacts in the crystal by the respective ‘random contact surfaces’ computed as if all the contact types occurred in an equiprobable way. The enrichment ratio is a novel and powerful tool derived from the Hirshfeld contact surface analysis to determine which type of contacts are over- or under-represented in crystal packings. A synthesis of the general tendencies found for the different molecular sets is shown in Table 2 ▶.

For instance, analysis of CHO- and CHN-type compounds has confirmed that H⋯O and H⋯N contacts are generally favoured as they form hydrogen bonds. H⋯H contacts are often found to have enrichment ratios close to or slightly lower than unity for the different kinds of molecules. However, in CHN and CHO aliphatic compounds H⋯H contacts are more under-represented in molecules with low hydrogen content, due to competition with hydrogen acceptors. The H⋯N and H⋯O contact enrichments tend to increase in aliphatic compounds when *S*_H_ becomes low; this is less the case in aromatic compounds where H atoms are also attracted by C atoms.

Aromatic compounds, which have significant amounts of carbon on their surface, can display C⋯C interactions that can be very enriched when they have heterocycles containing N, O, S or F atoms. Stacking between aromatic units is favoured when electron-rich aromatics interact with electron-deficient aromatics. The C⋯C contacts are particularly favoured, with *E*_CC_ ratios reaching values in the [2, 4] interval, in aromatic molecules which have a low or moderate proportion of hydrogen on their surface. However, it has to be remembered that in compounds with low *S*_C_, the occurrence of very large *E*_CC_ ratios is facilitated by numerical effects. When hydrogen is very abundant at the molecular surface, the higher propensity of C⋯H interactions to occur lowers as a consequence of C⋯C contacts.

In CH compounds C⋯C contacts are also more favoured when *S*_H_ is small, but the *E*_CC_ values are much smaller as π⋯π stacking is less favourable when there is no heterocycle.

The enrichment ratios are a useful tool which can be applied to specific classes of molecules to identify new relationships between their structure, crystal contacts and properties or point out some outliers. The most relevant non-covalent interactions (or synthons) in crystals are π⋯π stacking interactions and hydrogen bonds (Choprah & Row, 2011 ▶), which have been extensively studied in the literature and can display high enrichment ratios (H⋯O, C⋯C). For instance, correlations between different *E*_*XY*_ ratios could be discovered within families of molecules (Fig. 10 ▶). Crystal engineering consists of using the understanding of the formation of intermolecular interactions in crystal packings to design new solids with desirable chemical or physical properties (Desiraju, 1989 ▶). The formation of the crystal packing is a compromise between all the strong and weak interactions occurring between the neighbouring molecules. Analysis of crystal contact-forming propensities depending on the composition of the molecular surface by chemical elements gives knowledge which can contribute significantly to crystal engineering purposes. Enrichment ratios could also be computed for protein/ligand contact surfaces and yield interesting new information for the understanding of molecular recognition and drug design.

## Supplementary Material

Contact enrichment graphs and list of compounds studied from CSD. DOI: 10.1107/S2052252514003327/ed5001sup1.pdf

## Figures and Tables

**Table 1:** Example of Hirshfeld contact surfaces and derived ‘random contact’ and ‘enrichment ratios’ for the coumarin-102 crystal (Bibila Mayaya Bisseyou *et al.*, 2012 ▶) Values in italics at the top of the table are the data obtained from *CrystalExplorer*. The enrichment ratios were not computed when the ‘random contacts’ were lower than 0.9%, as they are not meaningful.

Atoms	H	C	N	O
H	*56.4*	–	Contacts	(%)
C	*19.9*	*2.3*	–	–
N	*0.9*	*0.0*	*0.0*	–
O	*19.5*	*0.5*	*0.5*	*0.0*
Surface %	76.6	12.5	0.7	10.3
H	58.6	Random	Contacts	(%)
C	19.1	1.6	–	–
N	1.1	0.2	0.0	–
O	15.7	2.6	0.1	1.1
H	0.96	–	–	Enrichment
C	1.04	1.5	–	–
N	0.8	/	/	–
O	1.24	0.20	/	0.0

**Table 2:** Recapitulation of the clear general tendencies of the enrichment ratios *E* for the different sets of molecules The variation (increase, decrease, stability) of *E* as a function of increasing percentage of hydrogen at the molecular surface, *S*_H_, is indicated by arrows.

Set	Aromatics		Aliphatics	
CH	*E*_HH_ ∼ 1	→	–	
	*E*_CH_ ∼ 1.1	→	–	
CHN	*E*_HH_ ∼ 0.9	→	*E*_HH_ < 1	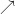
	*E*_NN_ < 1	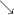	*E*_NN_ < 0.6	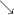
	*E*_HN_ > 1	→	*E*_HN_ > 1	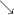
	*E*_CN_	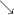	*E*_CN_	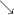
	*E*_HC_	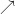	*E*_HC_	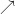
	*E*_CC_	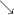	–	
CHO	*E*_HH_ ∼ 0.9	→	*E*_HH_	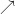
	*E*_OO_ < 0.6	→	*E*_OO_ < 1	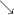
	*E*_HO_ > 1	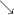	*E*_HO_ > 1	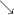
	*E*_CO_	→	–	
	*E*_HC_ < 1	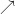	–	
CHS	*E*_HH_ ∼ 1	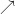	*E*_HH_ ∼ 1	→
	*E*_HS_ > 1	→	*E*_HS_	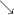
	*E*_CS_ < 1	–	–	
	*E*_HC_	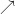	–	
	*E*_CC_	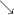	–	
CHF	*E*_HH_ < 1	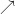	–	
	*E*_FF_ < 1	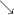	–	
	*E*_HF_ > 1	→	–	
	*E*_HC_	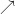	–	
	*E*_CF_	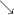	–	
