# Supplementary material for: The enrichment ratio of atomic contacts in crystals, an indicator derived from the Hirshfeld surface analysis
Source: IUCrJ. 2014 Feb 28;1(Pt 2):119–28. doi: 10.1107/S2052252514003327 (PMC4062089; doi:10.1107/S2052252514003327)
Supplement: Supplementary file 1 [file m-01-00119-sup1.pdf]

Supplementary Materials

The enrichment ratio of atomic contacts in crystal packings,
an indicator derived from the Hirshfeld surface analysis.

Christian Jelsch, Krzysztof Ejsmont, Loïc Huder,

Figure Sup1. Enrichment in CHN compounds as a function of nitrogen proportion S_N on the Hirshfeld surface. Left: Aromatic. Right: aliphatic, nitrile containing compounds.

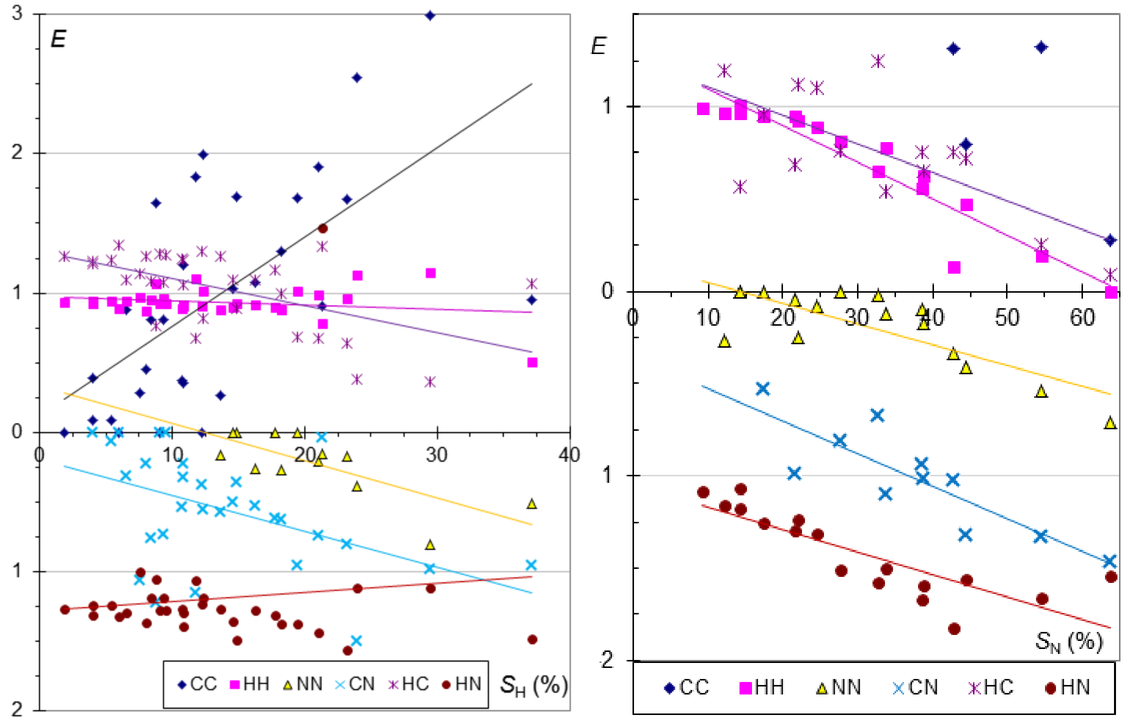

Figure Sup2. Enrichment of contacts in CHO aromatic compounds as a function of oxygen content on the Hirshfeld surface.

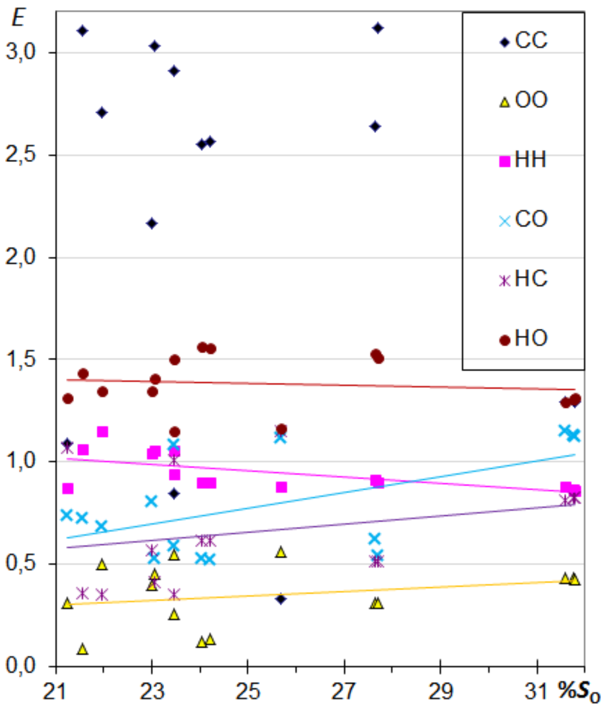

Figure Sup3. Contact enrichment ratios E_{OO} in crystals of CHO compounds as a function of % of oxygen on the Hirshfeld surface.

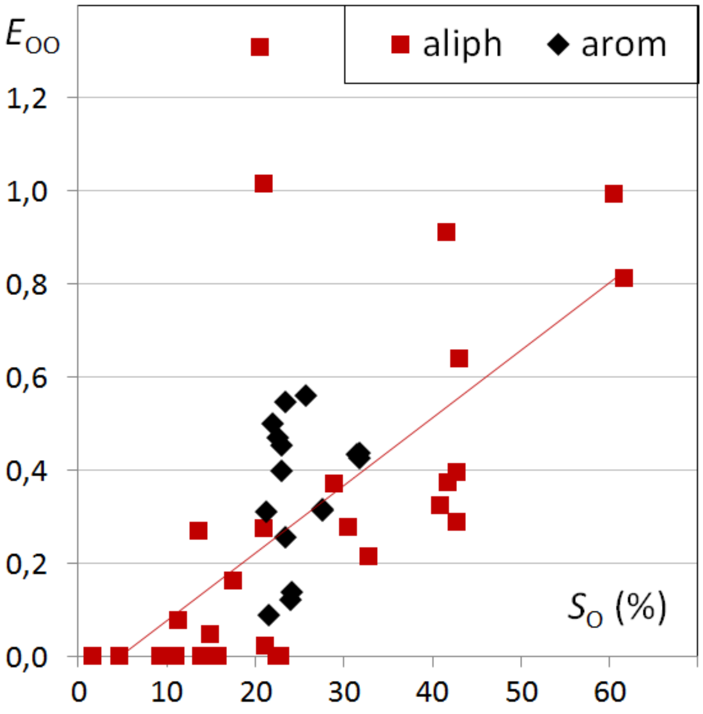

Figure Sup4. Enrichment in CHS aromatic compounds as a function of sulphur % on the Hirshfeld surface.

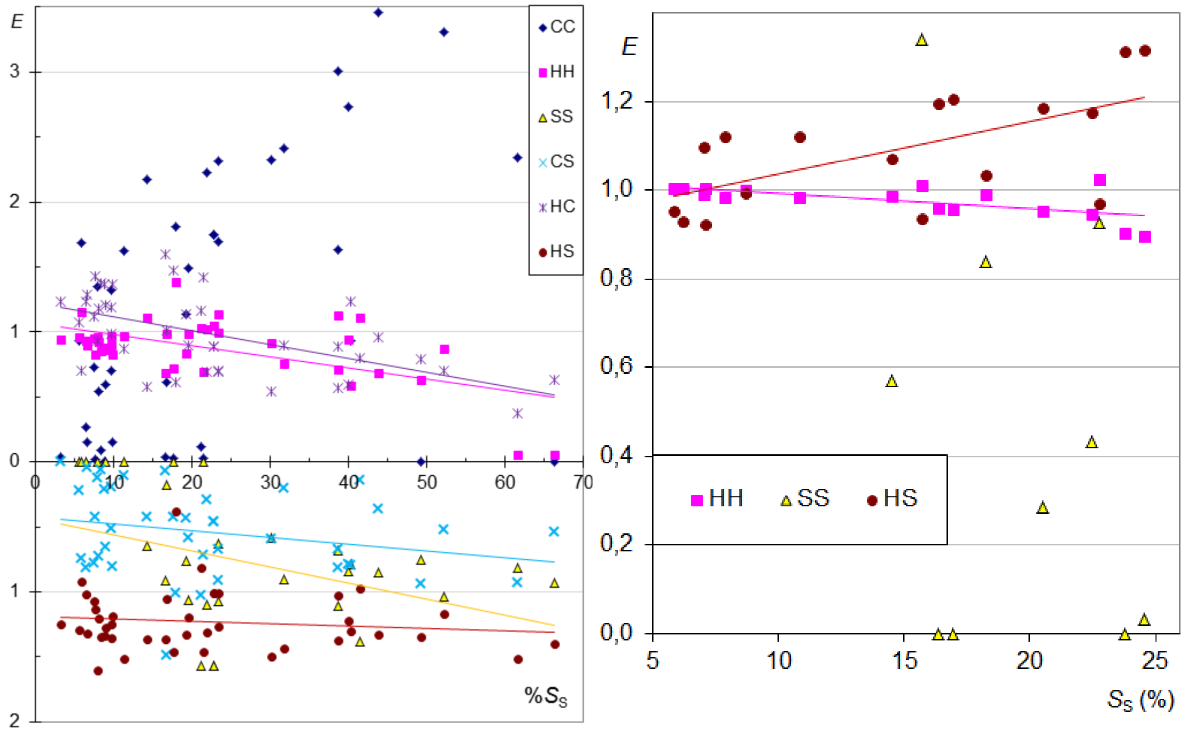

Figure Sup5. Enrichment of contacts in CHF compounds as a function of fluorine % on the Hirshfeld surface.

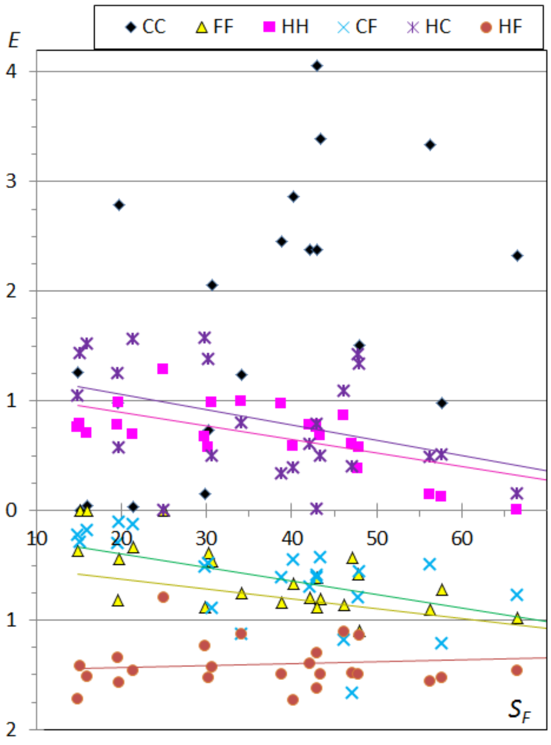

Table Sup1.

List of compounds retrieved from the Cambridge Structural Database included in the study.

LIST of CHN aromatic compounds.

AJOPOE01 ALACEV ADEMAX ADIVOZ AHELOO AROKUN AMEREQ AMERIU AWIXOT ARUDEW AYIJEX AJEXAP AHIMOU ACICAR ADIWEQ ATOBOA AWEHAL01 AJULAS APIVOL AGUXEF AMEPYD AMMEPY ADARAZ AFASOQ AMAPTZ

LIST of CHN aliphatic nitrile compounds.

METAMI HEMLAM FASGUB NAFHOS FIYCIZ HACRYN CYAMPD01 CALVAM BIGUAN01 ETDIAM01 BUFPAT CAACTY NEBQAM01 CEKGUU01 FAXHOB01 HAZGIY BISJIW DAMALN01 BITVUV DIBMEH DIBMIL HEXMDA01 BUGKIX01 ATCPEN01 CADVEI FIKHUC AWUDIF AZIBYR01 BUFGOY01 CAHVAI01 OFAGEI MAFZUP KUSVIE LIZBAX AMADAU

LIST of CHO aromatic compounds.

DETFOX ESCULT FUCOUM FUCOUN FURCOU HOHHOC IJUMEG IJUMEG01 LEZJAB LEZJAB01 PHGLOL WUYPOW ZZZEEU01 ZZZEEU08

LIST of CHO aliphatic compounds.

BASBIH BEQWEA AVOZIU BASBUT BASCAA CITQAY DIZWOY AVOZOA DEMDUV APTPEN10 AVOZUG BRASAC10 CIZGAT BUNVAH01 CUSCUO ACETAC01 CAFMIF ABEWAG ACRLAC02 BAHSUY CABBIQ01 CROTAC DGLYAC01 DLMALC11 CEZSEF ARABOL BASBON BKGLUT COTPAC DAYNUN ADIPAC04 BIVTUV01 BOMKUJ01 ALITOL01 DLMANT BUCACT01 CEBMOL CITRAC10 COWSUC AHEJAZ01 CEYLUN CEZSIJ DEQZUV AZELAC01

LIST of CHS aromatic compounds.

CEVZEJ COVTUC DBZTHP01 BIPHDS01 BOMZOS DIBTEO BZTBZT DOXTEP BIRKIW01 CEVZIN DIDGUT CIGLAF CELDUS DPHTPT10 DEQBUW DOYCEZ01 ABOJUX BATSAR BAFPIH01 CELDOM DESWED DESWAZ DESMIX DAWXOO ARIFIR BETMUI DABHET01 AFADOA DOXSUE DAFXIR CAZRIF CUZZAY

DAHDAR BUFQEY CIWJAU BOWWOZ BDTOLE10 BZPTHP DTPDTH DTHPDT BAHNUU01 DTENYL01

LIST of CHS aliphatic compounds.

UHOPAI ICAKIG PAGYIG PADQAN DEMQIW01 QEKXOT CEWKOF ICAKEC FEDQIP LIQWEN LIQWIR QEKXUZ HIYPAG QEKYAG WAZYUR QEKYEK RESGUR RESHAY TMETEY

LIST of CHF aromatic compounds.

KOYVOJ CAXNUL FLNAPH DFNAPH10 BAWPUK ATOZOY PFBIPH TFBIPH PUGPIQ FDPHET ASIJIV TEVLEM BANVES FACJAU CUWYUP PVVAWA01 PUGDEB FACGEV FACFOE FACFAQ
